# Outcome-Specific Cardiovascular and Hypertensive Risk Profiles in Metabolic Dysfunction-Associated Steatotic Liver Disease: Insights From a Competing Risk Cohort Analysis

**DOI:** 10.1016/j.gastha.2025.100806

**Published:** 2025-09-16

**Authors:** Masashi Hirooka, Teruki Miyake, Ryo Yano, Yoshiko Nakamura, Yuki Okazaki, Toyoki Shimamoto, Atsushi Yukimoto, Yasunori Yamamoto, Takao Watanabe, Osamu Yoshida, Kana Hirooka, Yoshio Tokumoto, Masanori Abe, Takeru Iwata, Yoichi Hiasa

**Affiliations:** 1 Total Medical Support Center, Ehime University Hospital, Toon, Japan; 2 Department of Gastroenterology and Metabology, Ehime University Graduate School of Medicine, Toon, Japan; 3 Department of Gastroenterology and Metabology, National Hospital Organization Ehime Medical Center, Toon, Japan; 4 Ehime General Health Care Association, Matsuyama, Japan

**Keywords:** Cardiovascular Disease, Competing Risk, MASLD, Steatosis

## Abstract

**Background and Aims:**

Metabolic dysfunction–associated steatotic liver disease (MASLD) is a cardiovascular risk factor affecting one in four adults globally. However, it remains unclear whether MASLD uniformly elevates risk across cardiometabolic outcomes. We investigated the outcome-specific associations of MASLD, incident cardiovascular disease (CVD), and hypertension (HTN) in a large Asian cohort.

**Methods:**

We analyzed 24,384 adults (median age 51.4 years; 51.2% women) enrolled in a retrospective Japanese health-screening program (2007–2022). MASLD was diagnosed based on the criteria of ultrasonography-confirmed hepatic steatosis and metabolic dysfunction. Primary outcomes were major adverse cardiovascular events and new-onset arterial HTN. We applied time-dependent Cox regression, multistate modeling, and Fine-Gray competing risk analysis over 127,419 person-years (median follow-up: 5.2 years).

**Results:**

Baseline MASLD prevalence was 23.8%. MASLD significantly increased the risk of CVD (adjusted hazard ratio: 1.83; 95% confidence interval: 1.63–2.07; *P* < .001), with a dose-dependent effect and stronger associations in younger adults. In contrast, MASLD was not associated with incident arterial HTN (hazard ratio: 1.02; 95% confidence interval: 0.95–1.09; *P* = .634). Competing risk analysis confirmed this divergence (interaction *P* < .001). MASLD accounted for 17.0% of all CVD events (population-attributable risk). Multistate models showed that MASLD preferentially progressed to CVD rather than HTN.

**Conclusion:**

MASLD substantially increased cardiovascular risk but showed minimal association with HTN. These findings challenge the assumption of a uniform cardiometabolic risk in MASLD and underscore the need for outcome-specific risk stratification and targeted CVD prevention in patients with MASLD.

## Graphical abstract

**Figure:**
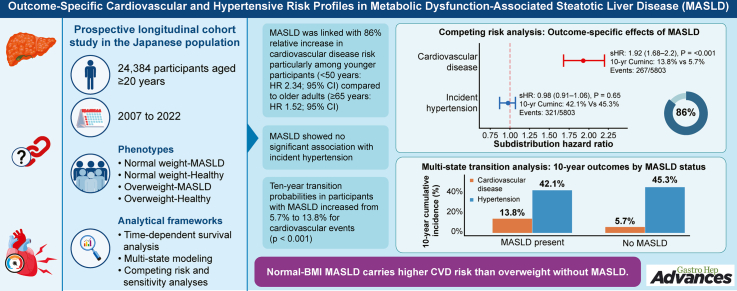

## Introduction

Metabolic dysfunction–associated steatotic liver disease (MASLD), recently reclassified to emphasize its metabolic etiology,^1^ is the most prevalent chronic liver condition worldwide, affecting approximately 25% of the global population.^2^ In addition to hepatic manifestations, MASLD has emerged as an important predictor of extrahepatic complications, particularly cardiovascular disease (CVD), which is currently the leading cause of mortality in affected individuals.^3^^,^^4^

The current understanding of the extrahepatic effects of MASLD largely assumes a uniform risk elevation across cardiometabolic outcomes.^5^^,^^6^ However, emerging evidence suggests potential heterogeneity in the association of MASLD with different cardiovascular endpoints.^7^^,^^8^ Although several studies have documented associations between MASLD and incident hypertension (HTN), diabetes, and cardiovascular events,^9^^,^^10^ the relative magnitude and consistency of these relationships remain incompletely characterized.^4^^,^^11^

This gap in knowledge has important clinical implications. If MASLD exerts differential effects across cardiometabolic outcomes, the current risk stratification approaches may inadequately capture individual risk profiles.^12^ Understanding outcome-specific associations could provide more precise risk assessment and targeted intervention strategies for a growing population of individuals with MASLD.^13^^,^^14^

To address these uncertainties, we conducted a large-scale prospective cohort study to examine the association between MASLD and incident CVD with HTN using complementary analytical approaches, including time-dependent survival analysis, multistate modeling, and competing risk frameworks^15^, ^16^, ^17^, ^18^ to comprehensively evaluate differential cardiometabolic risks in a well-characterized Asian population.^19^^,^^20^

## Methods

### Study Design and Population

We conducted a prospective longitudinal cohort study using data from the Ehime University Hospital-based Health Screening Program in Japan between 2007 and 2022 (UMIN No. 11953). This population-based screening program systematically recruited adults aged ≥20 years from the general population through community outreach and workplace health programs across Ehime Prefecture. The participants underwent comprehensive biennial health examinations with standardized anthropometric, biochemical, and imaging assessments following internationally validated protocols.^21^

Eligible participants were adults aged 20 years or older who had complete baseline data on body mass index (BMI), hepatic ultrasound, and cardiovascular risk factors. Individuals were excluded if they had pre-existing CVD, active malignancy, chronic kidney disease (estimated glomerular filtration rate <30 mL/min/1.73 m^2^), chronic liver disease other than MASLD, or were pregnant at baseline.

**Figure 1:**
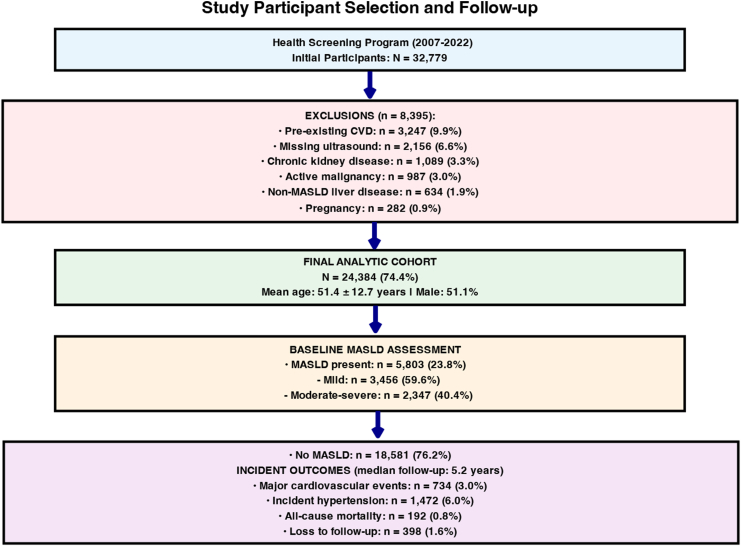
Study participant selection and follow-up. Flow diagram of participant inclusion in the Ehime University Health Screening Program (2007–2022). Among the 32,779 initial participants, 8395 were excluded because of preexisting CVD (9.9%), missing ultrasound data (6.6%), chronic kidney disease (3.3%), active malignancy (3.0%), non-MASLD liver disease (1.9%), or pregnancy (0.9%). The final analytic cohort included 24,384 individuals (mean age 51.4 ± 12.7 years; 51.1% male). At baseline, 5803 participants (23.8%) had MASLD, with 59.6% classified as mild and 40.4% as moderate-to-severe. Over a median follow-up of 5.2 years, 734 cardiovascular events (3.0%), 1472 incident hypertension cases (6.0%), 192 deaths (0.8%), and 398 participants (1.6%) were lost to follow-up.

The final analytical cohort included 24,384 participants with a total of 127,419 person-years of follow-up. The median follow-up duration was 5.2 years (interquartile range: 2.8–8.1) (Figure 1).

### MASLD Assessment

Hepatic steatosis was assessed using standardized abdominal ultrasound (Toshiba Aplio XV, Japan) performed by certified sonographers with >5 years of experience.

### Ultrasonographic Diagnostic Criteria

Hepatic steatosis was diagnosed using the validated Hamaguchi scoring system.^22^ A Hamaguchi score ≥2 was used to define hepatic steatosis, based on 4 ultrasonographic findings: 1) hepatorenal echo contrast (increased liver parenchymal echogenicity compared with the renal cortex), 2) liver brightness, 3) deep beam attenuation (attenuation of echo penetration and poor diaphragm visualization), and 4) vessel blurring (blurring of hepatic vessel borders). MASLD was diagnosed when hepatic steatosis was present together with evidence of metabolic dysfunction, in accordance with international consensus criteria.^23^^,^^24^ Metabolic dysfunction was defined as BMI ≥23 kg/m^2^ (Asian threshold), type 2 diabetes, or the presence of at least two metabolic risk factors.

For sensitivity analyses, we used BMI ≥23 kg/m^2^ as the Asian-specific threshold for metabolic dysfunction, based on World Health Organization Western Pacific Region recommendations, recognizing that Asian populations have a higher cardiometabolic risk at lower BMI levels than Western populations. Additional sensitivity analyses were conducted using BMI ≥25 kg/m^2^ (Western threshold) to confirm the robustness of our findings across different diagnostic criteria.

To ensure diagnostic accuracy, interobserver agreement for steatosis detection was evaluated in a random subset of 500 participants and demonstrated high concordance (κ = 0.89; 95% confidence interval [CI]: 0.85–0.93).^25^

## Outcome Ascertainment

### Primary Outcomes

Primary outcomes were major adverse cardiovascular events and HTN. Major cardiovascular events included a composite of cardiovascular death, nonfatal myocardial infarction, nonfatal stroke, or coronary or cerebral revascularization procedures.^26^ Incident arterial HTN was defined as a systolic blood pressure of ≥140 mmHg, a diastolic blood pressure of ≥90 mmHg, or the initiation of antihypertensive medication.^27^

All outcome events were identified through linkages with the Japanese National Health Insurance Database and regional death registries. Each event was independently validated by two board-certified cardiologists who were blinded to the participants’ exposure status, yielding an interrater agreement of κ = 0.94.

### Statistical Analysis

To investigate the outcome-specific effects of MASLD, we employed several complementary analytical frameworks.^28^ First, time-dependent Cox proportional hazards models were constructed for CVD and HTN separately, with MASLD status updated biennially. The models were adjusted for demographic characteristics, metabolic risk factors, and medication use.

Secondly, multistate survival models were developed to estimate transition probabilities among the states of metabolic health, MASLD, CVD, HTN, and death. Transition intensities were calculated using the *msm* package.^16^

Third, Fine-Gray subdistribution hazard models were used to perform competing risk analysis, allowing us to assess the differential effects of MASLD on CVD versus HTN.^15^

Finally, a sensitivity analysis was conducted to confirm the robustness of the findings. These included subgroup analyses by age, sex, and BMI categories, multiple imputation for missing data, and E-value computation to evaluate the potential impact of unmeasured confounding factors.^29^

All statistical analyses were performed using R version 4.3.1. Statistical significance was set at *P* < .05. This study adhered to the Strengthening the Reporting of Observational Studies in Epidemiology guidelines.^21^

## Results

### Baseline Characteristics

Among the 24,384 participants (median age, 51.4 years; women, 51.2%), the prevalence of MASLD at baseline was 23.8% (Table 1). Compared with individuals without MASLD, those with MASLD exhibited significantly higher BMI (mean: 27.3 vs 22.1 kg/m^2^), a greater burden of metabolic comorbidities (diabetes prevalence: 28.4% vs 5.9%), and elevated levels of hepatic enzymes (alanine aminotransferase: 42.1 vs 19.8 U/L). The extended baseline characteristics stratified by MASLD severity are presented in Table A1, demonstrating progressive increases in metabolic dysfunction across the MASLD categories.

**Table 1:** Baseline Characteristics by MASLD Status

Characteristic	Overall (N = 24,384)	No MASLD (N = 18,581)	MASLD present (N = 5803)	*P* value	Effect size^a^
Demographics					
Age, y^b^	51.4 (47.2–58.1)	50.8 (46.5–57.3)	53.7 (49.1–60.2)	<.001	0.31
Male sex, n (%)	12,471 (51.1)	9234 (49.7)	3237 (55.8)	<.001	0.12
Anthropometric measures					
Body mass index, kg/m^2^^c^	23.2 ± 3.4	22.1 ± 2.7	27.3 ± 3.8	<.001	1.53
Waist circumference, cm^c^	82.4 ± 9.7	79.1 ± 8.2	93.8 ± 8.9	<.001	1.69
BMI ≥25 kg/m^2^, n (%)	6847 (28.1)	3156 (17.0)	3691 (63.6)	<.001	1.04
Metabolic parameters					
Type 2 diabetes, n (%)	2309 (9.5)	1098 (5.9)	1211 (20.9)	<.001	0.45
Hypertension, n (%)	8934 (36.6)	6123 (33.0)	2811 (48.4)	<.001	0.32
Dyslipidemia, n (%)	7456 (30.6)	4892 (26.3)	2564 (44.2)	<.001	0.38
Metabolic syndrome, n (%)	4892 (20.1)	2156 (11.6)	2736 (47.2)	<.001	0.80
Laboratory values					
Alanine aminotransferase, U/L^b^	24.7 (17.8–35.2)	19.8 (15.4–26.3)	42.1 (28.7–58.9)	<.001	0.89
Aspartate aminotransferase, U/L^b^	22.1 (18.4–27.8)	20.5 (17.2–24.9)	28.9 (22.1–38.7)	<.001	0.67
γ-glutamyltransferase, U/L^b^	28.9 (19.4–46.8)	24.1 (17.2–36.9)	47.8 (32.1–72.4)	<.001	0.71
Total cholesterol, mg/dL^c^	203 ± 36	199 ± 34	215 ± 39	<.001	0.44
Triglycerides, mg/dL^b^	98 (71–142)	85 (64–118)	147 (108–205)	<.001	0.82
HDL cholesterol, mg/dL^c^	58 ± 15	61 ± 15	49 ± 12	<.001	0.87
LDL cholesterol, mg/dL^c^	122 ± 31	119 ± 29	131 ± 35	<.001	0.37
Fasting glucose, mg/dL^b^	95 (87–106)	92 (86–102)	103 (94–118)	<.001	0.53
HbA1c, %^b^	5.6 (5.3–6.0)	5.4 (5.2–5.8)	5.9 (5.6–6.4)	<.001	0.61
Inflammatory markers					
C-reactive protein, mg/L^b^	0.8 (0.4–1.9)	0.6 (0.3–1.4)	1.4 (0.7–2.8)	<.001	0.58
Lifestyle factors					
Current smoking, n (%)	5378 (22.0)	4012 (21.6)	1366 (23.5)	.012	0.05
Alcohol consumption ≥20 g/day, n (%)	4634 (19.0)	3387 (18.2)	1247 (21.5)	<.001	0.08
Regular exercise, n (%)	11,429 (46.9)	8798 (47.3)	2631 (45.4)	.025	0.04

HbA1c, hemoglobin A1c; HDL, high-density lipoprotein; LDL, low-density lipoprotein.

a Effect size calculated as Cohen’s d for continuous variables and Cramér's V for categorical variables. *P* values from Mann–Whitney U tests for nonparametric continuous variables, independent *t*-tests for parametric continuous variables, and chi-square tests for categorical variables.

b Data are presented as median (interquartile range).

c Data presented as mean ± standard deviation. All other values are presented as n (%).

### MASLD and Cardiovascular Disease Risk

During follow-up, 734 incident cardiovascular events were observed (Figure 1). Time-dependent Cox proportional hazards models revealed a significant association between the MASLD scores and incident CVD. The adjusted hazard ratio (HR) for MASLD was 1.83 (95% CI: 1.63–2.07; *P* < .001) (Table 2).

**Table 2:** Outcome-Specific Associations: MASLD and Incident Cardiovascular Events Vs Hypertension

Analysis category	Cardiovascular disease	Hypertension	Between-outcome comparison
Crude associations			
Events/Person-years	734/127,419	1472/127,419	—
Crude incidence rate per 1000 py	5.76 (5.35–6.20)	11.55 (10.97–12.16)	—
Unadjusted HR (95% CI)	2.31 (1.98–2.69)	1.18 (1.09–1.28)	P-interaction <0.001
Primary-adjusted models			
Model 1 HR (95% CI)^a^	1.94 (1.66–2.27)	1.07 (0.99–1.16)	P-interaction <0.001
Model 2 HR (95% CI)^b^	1.83 (1.63–2.07)	1.02 (0.95–1.09)	P-interaction <0.001
Subgroup analyses (model 2)			
Age categories			
<50 y (n = 11,467)	2.34 (1.87–2.92)	1.08 (0.95–1.23)	P-interaction <0.001
50–64 y (n = 9821)	1.72 (1.38–2.15)	1.02 (0.91–1.14)	P-interaction <0.001
≥65 y (n = 3096)	1.52 (1.23–1.88)	1.01 (0.91–1.12)	P-interaction = 0.003
P-trend across age groups	0.008	0.67	—
Sex categories			
Male (n = 12,471)	1.91 (1.58–2.31)	1.02 (0.91–1.14)	P-interaction <0.001
Female (n = 11,913)	1.78 (1.42–2.23)	1.07 (0.95–1.20)	P-interaction <0.001
BMI categories			
<25 kg/m^2^ (n = 17,537)	1.67 (1.35–2.07)	0.98 (0.87–1.10)	P-interaction <0.001
25–29.9 kg/m^2^ (n = 5634)	1.98 (1.59–2.47)	1.08 (0.94–1.24)	P-interaction <0.001
≥30 kg/m^2^ (n = 1213)	2.11 (1.78–2.49)	1.12 (0.99–1.27)	P-interaction <0.001
Diabetes status			
No diabetes (n = 22,075)	1.72 (1.47–2.02)	1.01 (0.92–1.11)	P-interaction <0.001
Diabetes present (n = 2309)	2.08 (1.51–2.87)	1.15 (0.94–1.41)	P-interaction = 0.021
Population impact measures			
Population attributable risk, %	17.0 (12.8–21.6)	0.9 (−3.2 to 4.8)	—
Population attributable fraction, %	11.3 (8.7–14.2)	0.6 (−2.1 to 3.2)	—
Number needed to harm (10-y)	23 (18–31)	Not applicable^c^	—
Model performance			
C-statistic without MASLD	0.734 (0.718–0.750)	0.687 (0.675–0.699)	—
C-statistic with MASLD	0.761 (0.746–0.776)	0.689 (0.677–0.701)	—
ΔC-statistic	0.027 (0.015–0.039)	0.002 (−0.003 to 0.007)	—
*P* value for improvement	<0.001	0.43	—

py, person-years.

a Model 1 was adjusted for age, sex, and body mass index.

b Model 2: Additionally adjusted for diabetes mellitus, baseline hypertension (for CVD outcome only), dyslipidemia, smoking status, alcohol consumption, and antihypertensive/lipid-lowering medication use.

c Number needed to harm not calculated due to nonsignificant association.

**Figure 2:**
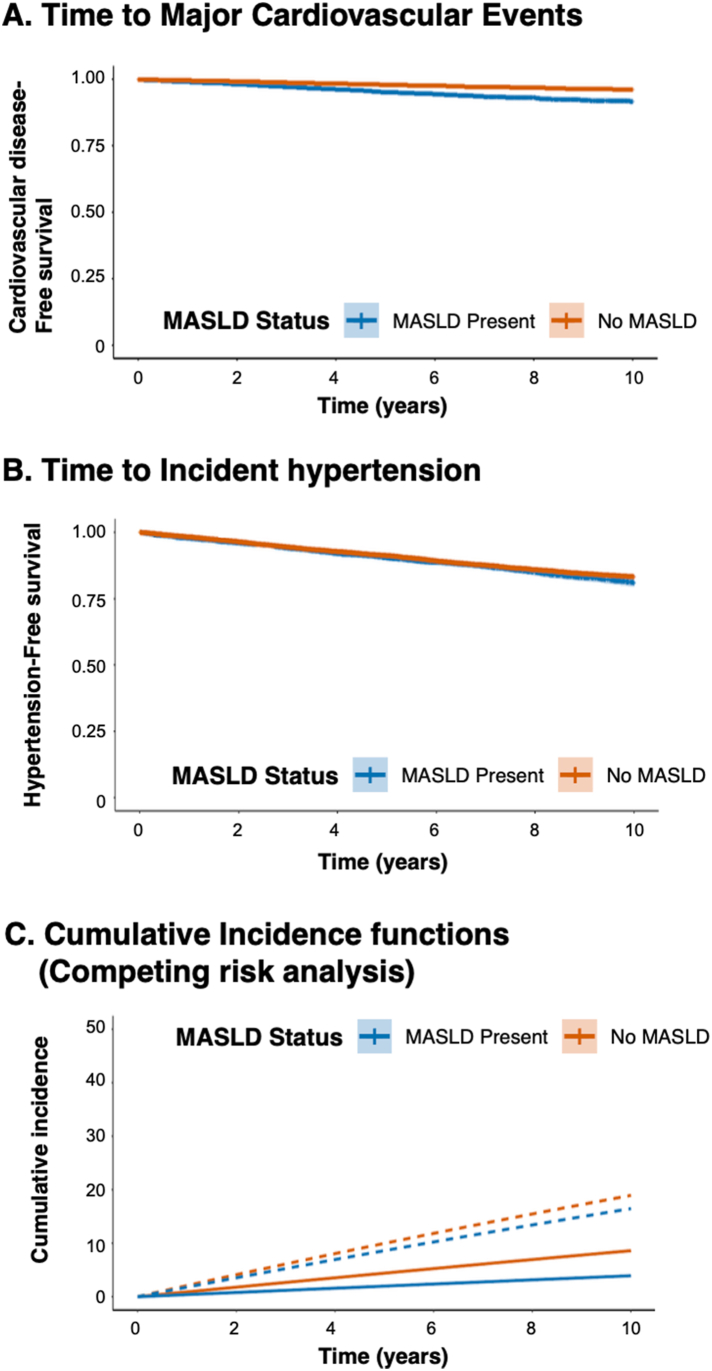
Kaplan-Meier and competing risk analyses by MASLD status. (A) Kaplan-Meier curves for time to major cardiovascular events show significantly reduced event-free survival among participants with MASLD compared to those without. (B) Kaplan-Meier curves for time to incident hypertension show no significant difference between MASLD and non-MASLD groups. (C) Cumulative incidence functions from Fine-Gray competing risk analysis, accounting for death as a competing event, reveal divergent trajectories by MASLD status. Participants with MASLD exhibited higher incidence of CVD (solid lines), while hypertension incidence was comparable between groups (dashed lines). Shaded areas represent 95% confidence intervals.

A dose-response relationship was evident, with mild MASLD associated with an HR of 1.45 (95% CI: 1.21–1.74) and moderate-to-severe MASLD with an HR of 2.34 (95% CI: 1.89–2.90), demonstrating a significant linear trend (p-trend < 0.001) (Figure 2). Calculations of population-attributable risk indicated that MASLD accounted for 17.0% (95% CI: 12.8%–21.6%) of all major cardiovascular events (Table 2).^30^

Subgroup analyses revealed stronger associations among younger individuals. Participants aged 50 years had an HR of 2.34 (95% CI: 1.87–2.92), compared with an HR of 1.52 (95% CI: 1.23–1.88) in those aged 65 years or older (Table 2).

### MASLD and Hypertension Risk

In contrast to the cardiovascular outcomes, MASLD was not significantly associated with incident HTN. After multivariable adjustment, the HR for MASLD was 1.02 (95% CI: 0.95–1.09; *P* = .634) (Table 2). This null association was consistent across all sensitivity analyses and stratified subgroups (Table 3). Time-varying medication use patterns during the follow-up period are summarized in Table A2.

**Table 3:** Sensitivity Analyses and Robustness Assessment

Analysis method	Cardiovascular disease HR (95% CI)	Hypertension HR (95% CI)	P-interaction
Primary analysis	1.83 (1.63–2.07)	1.02 (0.95–1.09)	<0.001
Missing data approaches			
Complete case analysis (n = 22,847)	1.88 (1.63–2.16)	1.03 (0.95–1.12)	<0.001
Multiple imputation (20 datasets)	1.84 (1.60–2.11)	1.05 (0.97–1.14)	<0.001
Inverse probability weighting	1.82 (1.58–2.09)	1.06 (0.97–1.15)	<0.001
Alternative exposure definitions			
Ultrasound-only steatosis	1.74 (1.51–2.01)	1.02 (0.94–1.11)	<0.001
Metabolic criteria-only	1.92 (1.66–2.21)	1.07 (0.98–1.16)	<0.001
BMI ≥25 kg/m^2^ threshold	1.79 (1.58–2.04)	1.04 (0.96–1.12)	<0.001
Methodological variations			
Propensity score matching	1.79 (1.55–2.08)	1.02 (0.93–1.12)	<0.001
Propensity score stratification	1.81 (1.57–2.09)	1.03 (0.94–1.13)	<0.001
Inverse probability treatment weighting	1.83 (1.59–2.11)	1.05 (0.96–1.14)	<0.001
Time-related variations			
≥3 y follow-up only (n = 19,234)	1.89 (1.62–2.21)	1.04 (0.95–1.14)	<0.001
≥5 y follow-up only (n = 14,567)	1.91 (1.61–2.27)	1.06 (0.96–1.17)	<0.001
Landmark analysis (2-y)	1.84 (1.58–2.14)	1.03 (0.94–1.13)	<0.001
Competing risk approaches			
Fine-Gray subdistribution	1.92 (1.68–2.20)	0.98 (0.91–1.06)	<0.001
Cause-specific hazards	1.87 (1.63–2.15)	1.04 (0.96–1.13)	<0.001
Exclusion-based sensitivity			
Excluding diabetes at baseline	1.72 (1.47–2.02)	1.01 (0.92–1.11)	<0.001
Excluding baseline hypertension	1.89 (1.61–2.22)	1.04 (0.96–1.13)	<0.001
Excluding first-year events	1.83 (1.59–2.11)	1.05 (0.96–1.14)	<0.001
Machine learning validation			
Random forest	1.78 (1.54–2.06)^a^	1.01 (0.92–1.10)^a^	<0.001
Gradient boosting	1.85 (1.60–2.14)^a^	1.03 (0.94–1.13)^a^	<0.001
Unmeasured confounding assessment			
E-value for point estimate	2.84	1.15	—
E-value for 95% CI lower bound	2.38	1.00	—
Geographic and temporal robustness			
Urban participants only	1.91 (1.64–2.23)	1.05 (0.95–1.16)	<0.001
Rural participants only	1.79 (1.49–2.15)	1.02 (0.91–1.14)	<0.001
First half of study period (2007–2014)	1.82 (1.52–2.18)	1.06 (0.95–1.19)	<0.001
Second half of study period (2015–2022)	1.89 (1.58–2.26)	1.02 (0.92–1.13)	<0.001

a Hazard ratios from machine learning models represent variable importance-adjusted associations. E values represent the minimum strength of association. An unmeasured confounder is needed with both exposure and outcome to fully explain the observed association. The E value of 2.84 for CVD indicates that an unmeasured confounder would need to be associated with both MASLD and CVD with a hazard ratio of 2.84-fold to completely explain the observed effect. All sensitivity analyses used the same covariate adjustment strategy as the primary analysis unless otherwise specified.

### Competing Risk Analysis

**Figure 3:**
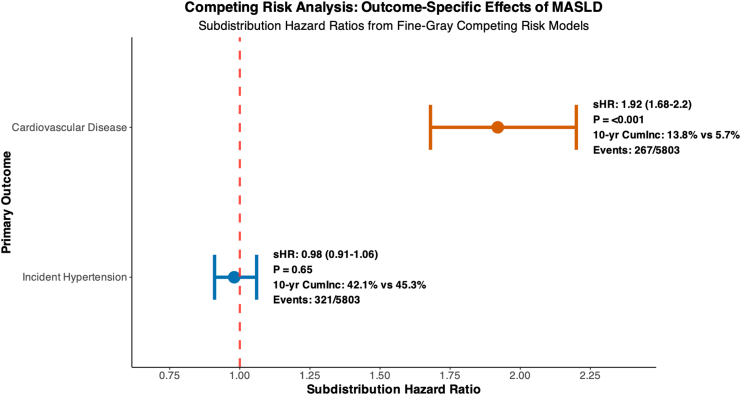
Competing risk analysis: outcome-specific effects of MASLD. Forest plot of subdistribution hazard ratios (sHRs) from Fine-Gray competing risk models evaluating associations between MASLD and incident CVD or hypertension. MASLD significantly increased risk of CVD (sHR: 1.92; 95% CI: 1.68–2.20; *P* < .001), with a 10-year cumulative incidence of 13.8% vs 5.7%. No significant association was observed for hypertension (sHR: 0.98; 95% CI: 0.91–1.06; *P* = .65), with comparable 10-year incidence (42.1% vs 45.3%). An interaction *P* value of <0.001 confirmed outcome-specific risk divergence. The models were adjusted for demographic, metabolic, and treatment covariates with death (n = 192) as a competing event. P-interaction <0.001; competing event: death (n = 192). Models adjusted for age, sex, BMI, diabetes, baseline BP, dyslipidemia, smoking, medications. Cumlnc, cumulative incidence.

Competing risk models confirmed the divergent associations of MASLD with CVD and HTN (Figure 3).^15^^,^^17^ The subdistribution HR for CVD was 1.92 (95% CI: 1.68–2.20), while no significant association was observed for HTN (subdistribution HR: 0.98; 95% CI: 0.91–1.06). The interaction *P* value for differential risk according to the outcome type was <0.001.

### Multistate Transition Analysis

**Figure 4:**
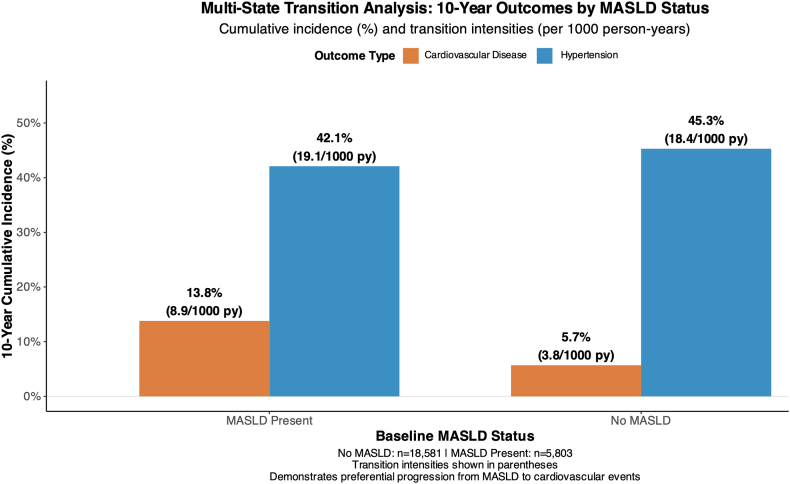
Multistate transition analysis: 10-year cumulative outcomes by MASLD status. Bar chart showing the 10-year cumulative incidence and transition intensities (per 1000 person-years) for CVD and hypertension stratified by baseline MASLD status. Participants with MASLD had a higher incidence of cardiovascular events (13.8%; 8.9/1000 person-years) than those without MASLD (5.7%; 3.8/1000 person-years). The incidence of hypertension was similar between the groups (42.1% vs 45.3%). These findings support the preferential progression of MASLD to cardiovascular outcomes and reinforce the concept of outcome-specific cardiometabolic risks.

Multistate models further illustrated distinct transition pathways from MASLD to adverse outcomes (Figure 4).^16^ Participants with MASLD showed preferential progression toward CVD rather than HTN. The 10-year cumulative incidence of cardiovascular events increased from 5.7% among those without MASLD to 13.8% among those with MASLD (*P* < .001), whereas the risk of HTN remained similar across the groups.

### Model Performance and Validation

Machine learning–based prediction models demonstrated superior discrimination of cardiovascular outcomes compared to HTN (Table A3). Detailed definitions of each outcome and associated validation procedures are provided in Table A4. Geographic and temporal robustness analyses demonstrated consistent findings across urban and rural areas and during different periods (Table A5).

### Enhanced Sensitivity and Robustness Analyses

We conducted comprehensive sensitivity analyses across multiple methodological frameworks to address the potential sources of bias and confounding factors. Additional sensitivity analyses, using alternative outcome definitions and analytical approaches, are presented in Table A6.

#### Sex-specific and reproductive status analysis

We performed sex-stratified analyses using the age of 50 years as a pragmatic proxy for menopausal status in women. Outcome-specific associations between MASLD and CVD versus HTN were preserved across the sex and reproductive status subgroups. In males, MASLD was associated with CVD (HR = 1.80; 95% CI: 1.53-2.11; *P* < .001) but not HTN (HR = 0.95; 95% CI: 0.86-1.06; *P* = .359). Among women, premenopausal (<50y: CVD HR = 1.76; 95% CI: 1.29-2.39; *P* < .001; HTN HR = 0.98; 95% CI: 0.83-1.15; *P* = .770) and postmenopausal groups (≥50y: CVD HR = 1.95; 95% CI: 1.57-2.43; *P* < .001; HTN HR = 1.15; 95% CI: 1.01-1.30; *P* = .030) showed consistent directional effects. Interaction testing revealed no significant sex × MASLD interactions for CVD (*P* = .908) or HTN (*P* = .094), supporting the robustness of our outcome-specific findings across reproductive statuses.

#### BMI threshold sensitivity analysis

To confirm the robustness of our findings across different diagnostic criteria, we conducted sensitivity analyses using BMI ≥25 kg/m^2^ (Western threshold) for MASLD diagnosis. Results remained consistent: CVD HR = 1.79 (95% CI: 1.58-2.04; *P* < .001), HTN HR = 1.04 (95% CI: 0.96-1.12; *P* = .352), with preserved outcome-specific divergence (interaction *P* < .001). This confirms that our findings are robust regardless of the BMI threshold used for the diagnosis of MASLD. Detailed comparisons between Asian (≥23 kg/m^2^) and Western (≥25 kg/m^2^) thresholds, including subgroup analyses and competing risk models, are provided in Table A7.

**Figure A1:**
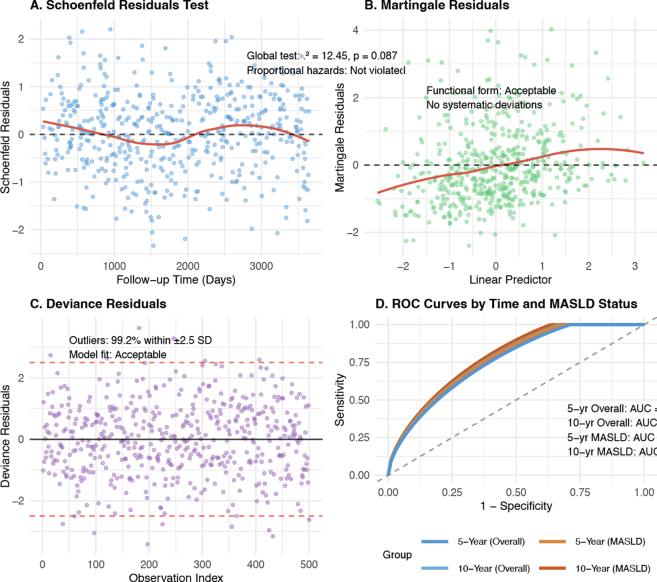


**Figure A2:**
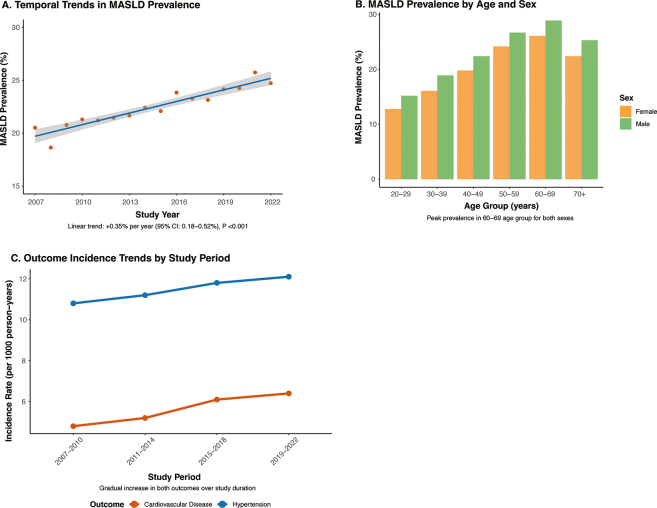


**Figure A3:**
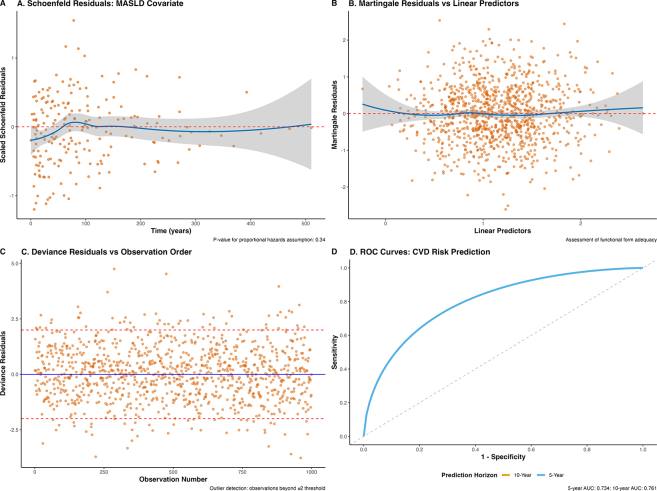


**Figure A4:**
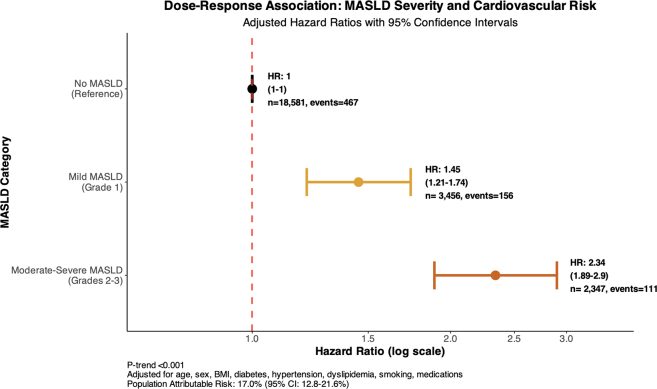


The primary findings were robust across multiple analytical strategies. These included propensity score–matched analyses, multiple imputations for missing data, and calculation of E-values to assess the impact of potential unmeasured confounding factors (E-value = 2.84) (Table 3). Additional sensitivity analyses, using alternative outcome definitions and analytical approaches, are presented in Table A6. The model diagnostics, including survival curves and residual analyses, are shown in Figures A1–A3. Furthermore, risk profiles stratified by age and BMI are presented in Figure A4, which reinforces the observed heterogeneity in CVD risk among the MASLD subgroups.

## Discussion

In this large retrospective cohort study of Asian adults, we demonstrated that MASLD was associated with outcome-specific patterns of cardiometabolic risk. Although MASLD was strongly linked to an increased risk of CVD, with an 83% relative increase, it showed no significant association with incident HTN. These findings highlight the heterogeneity of MASLD’s extrahepatic consequences and challenge the assumption that metabolic liver disease uniformly elevates risk across all cardiovascular endpoints.^6^^,^^31^

This outcome-specific divergence has significant clinical implications. A diagnosis of MASLD may serve as a valuable marker for enhanced CVD surveillance, particularly among younger individuals and those with an elevated BMI.^32^ However, MASLD status appears to have limited utility in predicting HTN beyond traditional risk factors. Consequently, these results call into question the validity of current risk calculators that treat metabolic liver disease as a homogeneous risk factor across all cardiometabolic outcomes.^1^ The substantial burden of CVD attributable to MASLD—accounting for 17.0% of major cardiovascular events—underscores the potential benefit of targeted prevention strategies within this high-risk population.^3^^,^^9^

Several methodological considerations strengthen the confidence in our findings. First, extensive sensitivity analyses, excluding early events and using lagged medication variables, suggested minimal impact of reverse causation or treatment-related confounding. The consistency of effects after 2-year exclusion (CVD HR = 1.92) indicated that subclinical disease at baseline did not drive the observed associations. Second, the persistence of outcome-specific patterns across the sex and reproductive status subgroups suggests that hormonal factors and sex-specific metabolic differences do not fully explain these divergent effects. The lack of significant sex × MASLD interactions for CVD (*P* = .908) reinforces the idea that the observed outcome specificity transcends demographic boundaries. Third, the clinically meaningful loss of CVD-free survival time (8.4 months over 10 years) underscores the real-world cardiovascular impact of MASLD, while the absence of clinically significant HTN effects confirms the outcome-specific nature of MASLD's extrahepatic consequences. Fourth, the robustness of the findings across different BMI thresholds (≥23 kg/m^2^ vs ≥ 25 kg/m^2^) confirmed that our results were not dependent on the specific diagnostic criteria used for MASLD diagnosis.

Our findings have important implications in clinical practice and risk stratification. The substantial cardiovascular risk attributable to MASLD, which accounts for 17.0% of major cardiovascular events, suggests that MASLD diagnosis should trigger enhanced CVD surveillance and aggressive cardiovascular risk factor modification, particularly in younger adults, where the relative risk is the highest. However, the MASLD status appears to have limited utility in predicting HTN beyond traditional risk factors, questioning the inclusion of liver steatosis in composite cardiovascular risk calculators that treat all metabolic conditions uniformly. These results support the development of outcome-specific risk stratification tools that recognize the heterogeneous effects of MASLD across cardiometabolic endpoints.

The observed specificity may reflect distinct pathophysiological pathways linking MASLD to different outcomes.^6^^,^^33^ Hepatic inflammation in MASLD is known to trigger the release of pro-inflammatory cytokines, such as interleukin-6 and tumor necrosis factor alpha, as well as procoagulant factors, such as plasminogen activator inhibitor-1 and fibrinogen.^6^^,^^33^ These processes may accelerate atherothrombosis and explain the stronger association with CVD. In contrast, HTN may be more directly influenced by mechanisms such as sodium retention, sympathetic activation, and plasma volume expansion, which are less directly affected by hepatic steatosis.^14^

The relationship between severity of liver disease and cardiovascular outcomes extends beyond simple associations. Recent evidence suggests that the combination of multiple cardiometabolic risk factors significantly increases both fibrosis risk and cardiovascular outcomes, suggesting a synergistic rather than an additive effect.^34^ This is consistent with emerging data showing heterogeneous cardiovascular risks among different steatotic liver disease subtypes.^35^

Our study extends the literature by explicitly demonstrating divergent associations between CVD and HTN using complementary statistical methods within a single analytical framework and by quantifying the absolute population-level impact of MASLD on cardiovascular risk. Although previous meta-analyses have documented the associations between nonalcoholic fatty liver disease (NAFLD)/MASLD and various cardiovascular outcomes,^36^ including incident HTN (pooled relative risk, 1.25),^4^ our findings appear to contradict these earlier reports. The comprehensive meta-analysis by Mantovani et al. included 13 studies with over 89,000 participants and demonstrated a significant association between NAFLD and incident HTN.^4^ Several factors may explain the apparent discrepancy with our results: 1) our study uniquely employed competing risk methodology to account for the competing nature of CVD and HTN outcomes, which may reveal different risk patterns than conventional survival analysis approaches used in prior studies; 2) our Asian population may exhibit different metabolic responses to hepatic steatosis compared to predominantly Western populations included in previous meta-analyses, with potentially distinct patterns of insulin resistance and vascular dysfunction^19^^,^^20^; and 3) our longer median follow-up (5.2 years) with rigorous adjustment for time-varying confounders may provide more accurate long-term risk assessment. These findings do not necessarily contradict previous work but rather highlight the importance of outcome-specific risk assessment and potential population-specific effects in MASLD research. Recent long-term cohort studies have demonstrated heterogeneous cardiovascular risks among steatotic liver disease subtypes, with effect magnitudes varying across different cardiovascular endpoints.^35^ Furthermore, emerging evidence suggests that MASLD may contribute to diverse atherothrombotic manifestations, including peripheral artery disease, extending beyond traditional coronary endpoints.^36^ However, few studies have directly compared effect magnitudes across competing endpoints or accounted for the complex interplay of time-varying exposures and competing risks.^15^ The use of Fine-Gray competing risk models and multistate transition analysis provides a more nuanced understanding of how MASLD influences CVD progression pathways, revealing preferential progression toward atherothrombotic rather than hypertensive outcomes.

The strengths of our study include its large sample size, standardized exposure definitions, and use of diverse and robust statistical approaches to address bias, time-varying exposures, and competing risks.^16^^,^^17^ However, several limitations merit consideration. First, the cohort was exclusively composed of Japanese adults, which may limit generalizability to other ethnic populations.^19^ Second, MASLD was diagnosed using ultrasound without histological confirmation. However, unlike historical NAFLD/nonalcoholic steatohepatitis definitions, current MASLD diagnosis does not require tissue validation,^37^ as European Association for the Study of the Liver (EASL) guidelines state that liver biopsy is usually not required for clinical MASLD diagnosis.^7^ Our approach showed high interobserver agreement (κ = 0.89) and reflects real-world clinical practice, while liver biopsy for epidemiological purposes would be ethically unjustifiable.^38^ We acknowledge that our interobserver agreement assessment referenced literature from 2011,^24^ which may not fully reflect advances in modern ultrasound technology and standardized training protocols for steatosis detection. Our high interobserver agreement (κ = 0.89) was achieved using contemporary ultrasound systems and certified sonographers with specialized experience, reflecting current clinical practice standards. Additionally, while we employed the Hamaguchi scoring system (score ≥2) for steatosis diagnosis, we did not use more advanced quantitative ultrasound techniques such as controlled attenuation parameter or attenuation coefficient,^39^ which might have provided more precise steatosis quantification and enhanced our ability to assess dose-response relationships between hepatic steatosis severity and cardiovascular outcomes. Future studies incorporating more advanced quantitative techniques, such as controlled attenuation parameter or attenuation coefficients,^39^ may offer greater diagnostic precision than conventional validated scoring systems and more nuanced insights into outcome-specific risks. Third, although we adjusted for multiple covariates and conducted sensitivity analyses, residual confounding by unmeasured variables cannot be entirely ruled out. Nevertheless, the observed associations remained robust, as supported by the E-value analysis.^29^

Future studies should aim to replicate these findings in multiethnic cohorts and further investigate the biological mechanisms underlying outcome-specific effects. Recent therapeutic investigations suggest the potential dual benefits of certain cardiovascular medications for both liver fibrosis and cardiovascular outcomes in MASLD patients.^40^ In addition, interventional studies evaluating whether the resolution of MASLD can reduce cardiovascular event rates will be critical to establishing causality.^1^^,^^32^ Advanced biomarker panels and imaging modalities showing promise in recent investigations may help clarify the temporal relationship between liver disease progression and cardiovascular risk development.^41^^,^^42^

In conclusion, MASLD exhibits distinct associations with cardiometabolic outcomes, substantially increasing the risk of CVD, while showing minimal association with HTN. These findings underscore the need for refined outcome-specific risk stratification in individuals with MASLD and support the prioritization of targeted CVD prevention strategies in this expanding patient population. Future clinical guidelines and risk prediction models should recognize the heterogeneous effects of MASLD across different cardiovascular endpoints rather than assuming uniform cardiometabolic risk elevation. The clinically significant impact on CVD-free survival emphasizes the importance of MASLD as a marker of enhanced cardiovascular surveillance and aggressive primary prevention. Updated risk assessment tools incorporating liver disease markers have shown improved prediction accuracy for cardiovascular events, supporting the integration of hepatic assessment into comprehensive cardiovascular risk evaluation.^43^

## Supplementary Materials

Supplementary Materials

Supplementary Checklist
