# Supplementary material for: Outcome-Specific Cardiovascular and Hypertensive Risk Profiles in Metabolic Dysfunction-Associated Steatotic Liver Disease: Insights From a Competing Risk Cohort Analysis
Source: Gastro Hep Adv. 2025 Sep 16;5(1):100806. doi: 10.1016/j.gastha.2025.100806 (PMC12589983; doi:10.1016/j.gastha.2025.100806)
Supplement: Supplementary Materials [file mmc1.docx]

Clinical Study Protocol

Version: 3.0

Date: February 10, 2025

Title: Investigation of Factors Associated with Lifestyle-related Diseases in Health Checkup Examinees

Principal Investigator: Teruki Miyake, MD, PhD

Institution: Department of Gastroenterology and Metabology, Ehime University Graduate School of Medicine, Toon, Japan

# 1. Background and Objectives

In recent decades, the increasing prevalence of lifestyle-related diseases, including metabolic syndrome, type 2 diabetes mellitus, dyslipidemia, hypertension, hyperuricemia, and nonalcoholic fatty liver disease (NAFLD), has become a global concern. These conditions are closely linked to Westernized dietary habits, physical inactivity, smoking, poor sleep, and stress.

NAFLD, now more appropriately classified as metabolic dysfunction-associated steatotic liver disease (MASLD), can progress to steatohepatitis, cirrhosis, liver failure, and hepatocellular carcinoma. Furthermore, these conditions are known risk factors for atherosclerotic cardiovascular diseases (ASCVD).

This study aims to identify clinical, biochemical, and imaging-based predictors of lifestyle-related diseases and ASCVD using anonymized data obtained from routine health checkups conducted in a large Japanese cohort. Additionally, longitudinal data from participants who underwent multiple checkups will be used to explore predictors of disease onset over time.

# 2. Study Population

Study 1 (Cross-sectional analysis): Individuals who underwent health checkups at the Ehime Health Promotion Center between April 2003 and March 2025.
Study 2 (Retrospective longitudinal analysis): Individuals who received multiple health checkups during the same period.
Inclusion Criteria: Age ≥18 years; Attendees of the above health center during the study period.

# 3. Study Design and Methods

Study 1: Cross-sectional Study
Anonymized data from examinees will be analyzed to identify factors associated with the presence of lifestyle-related diseases, including MASLD, impaired glucose tolerance, dyslipidemia, hypertension, hyperuricemia, metabolic syndrome, and ASCVD.

Study 2: Retrospective Cohort Study
Among individuals with repeated checkup data, we will assess temporal changes in clinical parameters and identify predictors of incident disease outcomes.

Neural Network Model Development and Validation
A subset of the anonymized dataset will be used to train a feedforward neural network model for the prediction of hepatic steatosis (moderate-to-severe, as defined by ultrasound). As the neural network model will be developed using de-identified data with no re-identification possible, this AI-based analysis is covered under the same ethical framework and waiver of consent as the rest of the study. The methodology and findings will be published transparently in accordance with TRIPOD-AI guidelines.

Data Items Collected:
- Medical history and lifestyle questionnaires
- Anthropometric data (e.g., BMI, abdominal circumference)
- Blood pressure
- Blood tests: CBC, liver function tests (AST, ALT, ALP, γ-GTP, bilirubin, albumin, PT), lipid profile (total cholesterol, LDL, HDL, triglycerides), HbA1c, uric acid, HBs antigen, HCV antibody
- Urinalysis: proteinuria, occult blood
- Abdominal ultrasound: presence or absence of hepatic steatosis

# 4. Potential Risks and Safety Measures

This study uses data obtained during routine health checkups. No additional procedures will be performed. No adverse events are anticipated.

# 5. Ethical Considerations

All data used in this study are anonymized and non-linkable prior to analysis. Therefore, individual informed consent is waived under national ethical guidelines. A summary of the study has been disclosed publicly on the institutional website to ensure transparency.

This study complies with the Declaration of Helsinki, the Ethical Guidelines for Medical and Health Research Involving Human Subjects in Japan, and will be conducted under the oversight of the Ethics Committee of Ehime University Graduate School of Medicine (Approval No: to be provided).

# 6. Cost and Compensation

As this is a retrospective study based on pre-existing data, no additional costs will be incurred by participants, nor will any financial compensation be provided.

# 7. Data Management and Confidentiality

All anonymized datasets will be securely stored by the principal investigator. The data will be retained for a period of 5 years after the completion of the study, after which they will be permanently deleted.

# 8. Publication and Dissemination of Results

The findings will be disseminated through presentations at academic conferences and publications in peer-reviewed journals, including Gastroenterology. The data will not be used for any other purpose.

# 9. Research Organization and Collaborating Institutions

Principal Investigator:
- Teruki Miyake, MD, PhD – Department of Gastroenterology and Metabology, Ehime University

Collaborating Institution:
- Ehime Health Promotion Center

# 10. Funding and Conflicts of Interest

This study is investigator-initiated and not funded by any commercial entity. The authors declare no conflicts of interest.

# 11. References

1. Miyake T, Kumagi T, Hirooka M, et al. J Gastroenterol. 2015
2. Miyake T, et al. J Gastroenterol. 2015
3. Miyake T, et al. J Gastroenterol. 2016
