# Supplementary material for: Outcome-Specific Cardiovascular and Hypertensive Risk Profiles in Metabolic Dysfunction-Associated Steatotic Liver Disease: Insights From a Competing Risk Cohort Analysis
Source: Gastro Hep Adv. 2025 Sep 16;5(1):100806. doi: 10.1016/j.gastha.2025.100806 (PMC12589983; doi:10.1016/j.gastha.2025.100806)
Supplement: Supplementary Checklist [file mmc2.docx]

**STROBE Checklist for Cross-sectional Studies**

**The cardiovascular risk paradox: normal-weight individuals with steatotic liver disease are at markedly elevated cardiovascular risk beyond BMI-based detection"**

| **Item** | **Section/Topic** | **Recommendation** | **Page/Line** | **Status** |
| --- | --- | --- | --- | --- |
| **1** | **Title and abstract** |  |  |  |
| 1(a) | Title | Indicate the study's design with a commonly used term in the title or the abstract | Page 1, lines 1-3 | ✓ Cross-sectional design |
| 1(b) | Abstract | Provide in the abstract an informative and balanced summary of what was done and what was found | Page 2, lines 1-15 | ✓ Complete structured abstract |
| **2** | **Introduction** |  |  |  |
| 2 | Background/rationale | Explain the scientific background and rationale for the investigation being reported | Page 2, lines 19-Page 3, lines 11; | ✓ BMI-centric limitations explained |
| 3 | Objectives | State specific objectives, including any prespecified hypotheses | Page 3, lines 12-19 | ✓ Hypothesis clearly stated |
| **3** | **Methods** |  |  |  |
| 4 | Study design | Present key elements of study design early in the paper | Page 2, lines 4 | ✓ Cross-sectional design described |
| 5 | Setting | Describe the setting, locations, and relevant dates | Page 10, lines 23-27 | ✓ Japan, 2007-2010 specified |
| 6 | Participants | Give the eligibility criteria, and the sources and methods of selection of participants | Page 10, lines 27-30 | ✓ Inclusion/exclusion criteria |
| 7 | Variables | Clearly define all outcomes, exposures, predictors, potential confounders, and effect modifiers | Page 10, lines 35-Page 11, lines 28 | ✓ NWSLD, CVD, covariates defined |
| 8 | Data sources/measurement | For each variable of interest, give sources of data and details of methods of assessment | Page 11, lines 12-28 | ✓ FLI, clinical measures described |
| 9 | Bias | Describe any efforts to address potential sources of bias | Page 9, lines 5-19 | ✓ Non-differential misclassification |
| 10 | Study size | Explain how the study size was arrived at | Page 10, lines 27-30 | ✓ Population-based screening |
| 11 | Quantitative variables | Explain how quantitative variables were handled in the analyses | Page 11-12, lines 30-33 | ✓ Continuous/categorical handling |
| 12 | Statistical methods | Describe all statistical methods, including those used to control for confounding | Page 11-12, lines 25-19 | ✓ Logistic regression, ML |
| 12(a) | Statistical methods | Describe any methods used to examine subgroups and interactions | Page 12, lines 3-16 | ✓ Subgroup analyses |
| 12(b) | Statistical methods | Explain how missing data were addressed | Page 12, lines 16-17 | ✓ Multiple imputation |
| 12(c) | Statistical methods | Describe any sensitivity analyses | Page 12, lines 15-17; Page 6, lines 5-15 | ✓ FLI thresholds, BMI cutoffs |
| **4** | **Results** |  |  |  |
| 13 | Participants | Report numbers of individuals at each stage of study and reasons for non-participation | Page 3, lines 23-24; Page 10, lines 27-30 | ✓ Flow described in text |
| 14 | Descriptive data | Give characteristics of study participants and information on exposures and potential confounders | Page 3, Lines 24-30, Table 1 | ✓ Baseline characteristics |
| 15 | Outcome data | Report numbers of outcome events or summary measures | Page 4, lines 3-7 | ✓ CVD events by phenotype |
| 16 | Main results | Give unadjusted estimates and confounder-adjusted estimates and their precision | Page 4, lines 8-23 | ✓ Crude and adjusted ORs |
| 16(a) | Main results | Report category boundaries when continuous variables were categorized | Page 11, lines 8-10; Page 11, lines 13-15 | ✓ BMI <25 vs ≥25 kg/m² |
| 16(b) | Main results | If relevant, consider translating estimates of relative risk into absolute risk for a meaningful time period | Page 4, lines 16-17; Page 5, lines 23-35 | ✓ NNT=34, absolute risks |
| **5** | **Discussion** |  |  |  |
| 17 | Key results | Summarise key results with reference to study objectives | Page 6, lines 18-24 | ✓ Key findings summarized |
| 18 | Limitations | Discuss limitations of the study, taking into account sources of potential bias or imprecision | Page 9, lines 2-19 | ✓ Cross-sectional design, generalizability |
| 19 | Interpretation | Give a cautious overall interpretation of results considering objectives, limitations, multiplicity of analyses, results from similar studies, and other relevant evidence | Page 6-8, lines 25-6 | ✓ Balanced interpretation |
| 20 | Generalisability | Discuss the generalisability (external validity) of the study results | Page 8, lines 10-20; Page 9, lines 13-16 | ✓ Asian population, screening settings |
| **6** | **Other information** |  |  |  |
| 21 | Funding | Give the source of funding and the role of the funders for the present study | Page 13, lines 5-7 (Acknowledgments) | ✓ Support mentioned |
